# Assessing Local and Surrounding Threats to the Protected Area Network in a Biodiversity Hotspot: The Hengduan Mountains of Southwest China

**DOI:** 10.1371/journal.pone.0138533

**Published:** 2015-09-18

**Authors:** Xin Ye, Guohua Liu, Zongshan Li, Hao Wang, Yuan Zeng

**Affiliations:** 1 State Key Laboratory of Urban and Regional Ecology, Research Center for Eco-environmental Sciences, Chinese Academy of Sciences, Beijing, China; 2 University of Chinese Academy of Sciences, Beijing, China; 3 Institute of Remote Sensing and Digital Earth, Chinese Academy of Sciences, Beijing, China; Chinese Academy of Forestry, CHINA

## Abstract

Protected areas (PAs) not only serve as refuges of biodiversity conservation but are also part of large ecosystems and are vulnerable to change caused by human activity from surrounding lands, especially in biodiversity hotspots. Assessing threats to PAs and surrounding areas is therefore a critical step in effective conservation planning. We apply a threat framework as a means of quantitatively assessing local and surrounding threats to different types of PAs with gradient buffers, and to main ecoregions in the Hengduan Mountain Hotspot of southwest China. Our findings show that national protected areas (NPAs) have lower and significantly lower threat values (p<0.05) than provincial protected areas (PPAs) and other protected areas (OPAs), respectively, which indicates that NPAs are lands with a lower threat level and higher levels of protection and management. PAs have clear edge effects, as the proportion of areas with low threat levels decline dramatically in the 5-kilometer buffers just outside the PAs. However, NPAs suffered greater declines (58.3%) than PPAs (34.8%) and OPAs (33.4%) in the 5-kilometer buffers. Moreover, a significant positive correlation was found between the size of PAs and the proportion of areas with low threat levels that they contained in both PAs and PA buffers (p<0.01). To control or mitigate current threats at the regional scale, PA managers often require quantitative information related to threat intensities and spatial distribution. The threat assessment in the Hengduan Mountain Hotspot will be useful to policy makers and managers in their efforts to establish effective plans and target-oriented management strategies.

## Introduction

Protected areas (PAs) are a cornerstone of biodiversity conservation efforts, as they provide various species with safe havens [1]. However, the rapid increase in human land use surrounding PAs gradually diminishes these protected areas’ capacity to preserve species and maintain ecological processes [2, 3]. Habitat loss and habitat isolation are two direct consequences of threats both within and surrounding their PA boundaries [4–6]. Therefore, PAs may not be effective when they fail to limit habitat loss and are unconnected via corridors to other wild areas. Among those threats, human activities such as expanding settlement, agricultural activities, and road construction may cause further serious threats by reducing the effective size of PAs, damaging ecosystem services, and increasing exposure at PA edges [3, 7–10].

An ideal PA network should contain not only the habitats of core areas within PAs, but also habitats of buffer zones in the surrounding areas with relatively few human activities and stable land use types, which could encourage connections between habitat patches, movement of species, and nutrient and energy flows [11, 12]. In addition to preserving biodiversity, PAs should maintain natural processes and promote survival of species by excluding threats [13]. To achieve these goals, we must understand what the main threats are, where the potential threats occur, and where high-risk areas are distributed. Identifying these threats is therefore crucial for conservation managers to take effective measures to mitigate some of the proximate threats to PAs [14]. However, a critical limitation in our current knowledge is an understanding of how human activities and increasing threat intensity inside PAs and outside PAs can reduce the effectiveness of PAs across PA networks [15, 16]. Hence, assessing threats at regional scales is a prerequisite for analyzing and predicting ecological consequences. Several studies have assessed the vulnerability related to threatening processes using different types of methods, including methods based on coverage of existing PAs [17], methods based on the numbers of threatened species and threat ratings [18], and methods based on expert opinions [19]. Spatially mapped threatening processes based on spatial variables and environmental characteristics are more convenient for conservation planners seeking to execute targeted decisions [20, 21]. In addition, more studies on monitoring and evaluating the status and changes of natural ecosystems and the pattern of human activities have paid more attention to PA networks at regional scales [22–24]. In short, prioritizing future conservation planning at regional scales must consider the threats to both protected areas and adjacent land use that may impact to a conservation system.

Conservation International’s Biodiversity hotspots, which have particularly high endemic species richness and face extreme threats of high levels of habitat destruction, are priority areas critical to biodiversity conservation at a global scale [25–29]. However, hotspots only retain 14.9% of their area as natural intact vegetation (NIV), and most have suffered NIV losses and are thought to contain less NIV than previously estimated [30]. The Mountains of Southwest China Hotspot, the central area of the Hengduan Mountains, is one of 35 hotspots in the world. As one of the richest temperate floristic areas in the world, the region is well known for its biodiversity [31]. Despite its relatively small population due to the region’s extreme topography and climate, the Mountains of Southwest China Hotspot is nevertheless heavily impacted by human activities. With the rapid economic development in China, more and more roads have been constructed as part of the Great Western Development Strategy [32, 33]. In addition, high population growth rates and the rapid development of tourism have exacerbated pressures on natural habitats. Although the Chinese government has implemented several natural protection and ecological restoration projects, such resource extraction activities as logging and the collection of medical plants and animals have never ceased, especially upon completion of these protection and restoration projects. Hence, assessing the number and intensity of potential threats to this biodiversity hotspot is of the utmost importance to maintaining regional biodiversity and the effectiveness of protected areas.

Most PAs do not exist as isolated habitats but are surrounded by a large matrix of human disturbance. The surrounding matrix not only provides continuous habitat corridors to other PAs but also contexts for assessing the threats that PAs face [34]. However, most of the studies conducted in the Mountains of Southwest China Hotspot have been based only on a single protected area. There is a shortage of comprehensive studies examining PA networks and the buffers surrounding them. Moreover, the existing studies on PA threat analysis in this hotspot have mostly focused on qualitative description or quantitative analysis without specific space information. Here, we used high precise land cover data and detailed information regarding transportation networks to assess the potential threats to PAs and their buffers around them. We compared the threats between protected and unprotected areas in different ecoregions, and among PAs under different protection levels, and then assessed relationships between PA sizes and threat levels. Our aim is to provide an assessment of the PA network in the Mountains of Southwest China Hotspot that directly incorporates the PA and surrounding threats in order to identify PA effectiveness and determine where highly-threatened habitats exist. We anticipate that the assessment will be able to provide tools for PA managers to make effective conservation and restoration decisions. Overall, the study and conservation of the Mountains of Southwest China Hotspot will have an important influence on global biodiversity conservation.

## Materials and Methods

### Study area

**Fig 1:**
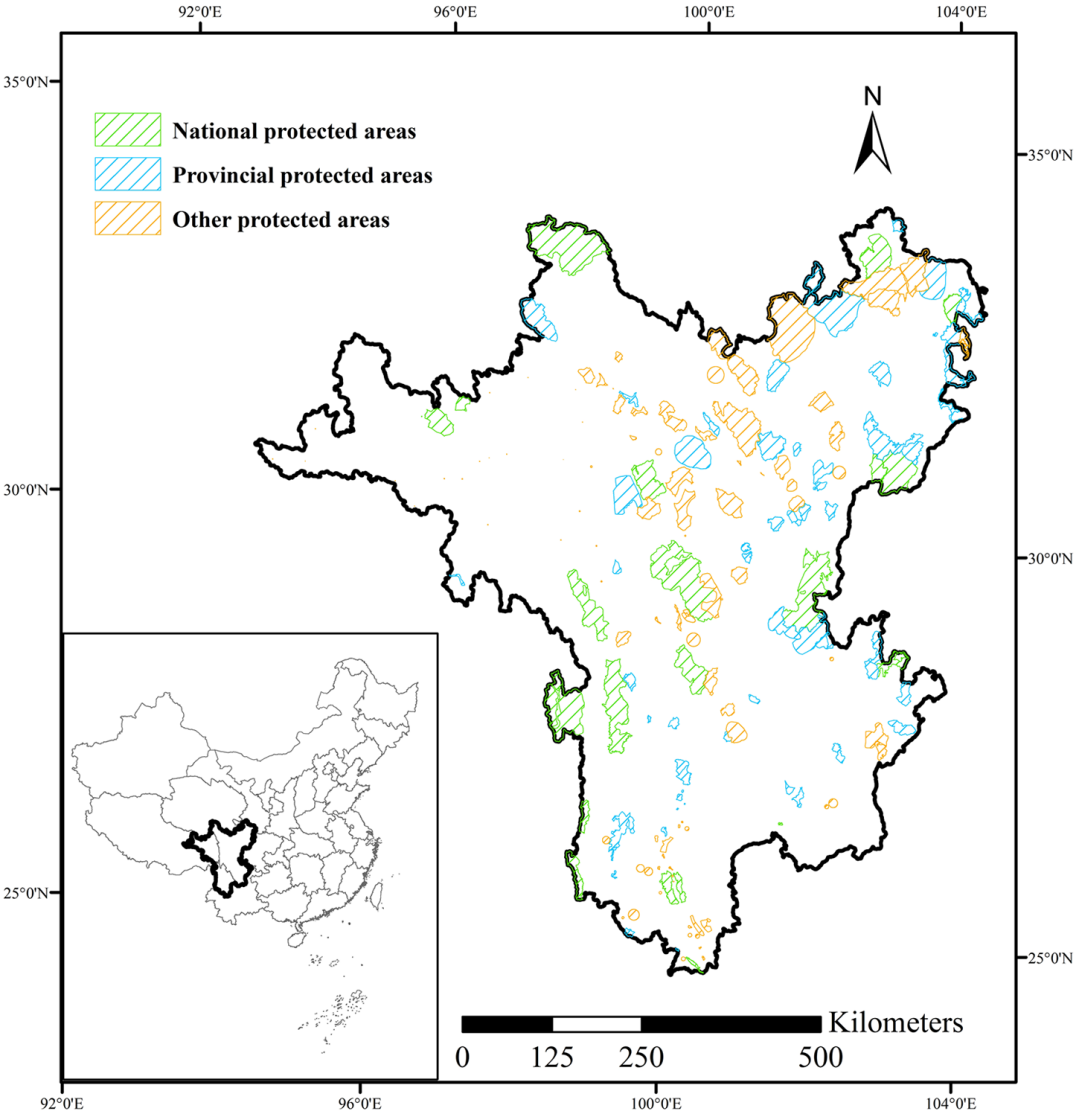
Location of the Hengduan Mountain Hotspot and spatial distribution of 159 protected areas according to three constructed administrative grades: national protected areas, provincial protected areas, and other protected areas.

The Hengduan Mountains are located in southwest China, most of which was covered by the Global Biodiversity Conservation Priorities and Global Biodiversity Hotpots [27, 30, 35], and was referred to as the Hengduan Mountain Hotspot in this study (Fig 1). There are three Global 200 priority ecoregions (G200s) in this region, namely, the Tibetan Plateau Steppe in the north, the Hengduan Mountain Conifer Forests in the middle, and the North Indochina Subtropical Moist Forests in the south. The Mountains of Southwest China, situated in the central Hengduan Mountains, is one of 35 hotspots in the world. The region contains the upstream regions of such major rivers as the Yellow River, the Yangtze River, and the Lancang River, and is a treasure house of species. With dramatic variations in topography, different areas varying in elevation by over 6,000 meters, the Hengduan Mountain Hotspot supports a wide array of habitats, including the richest temperate endemic flora worldwide. The total land area of the Hengduan Mountain Hotspot amounts to approximately 493,334 km^2^. Coniferous forest comprises nearly 23% of the hotspot’s total area, and meadows and steppes comprise nearly 32% of its total area.

Rapid economic and population growth have made the reservoirs of plant and animal life in this hotspot among the richest but most highly threatened. Examples of threatened species include *Rhinopithecus roxellana*, *Ailuropoda melanoleuca*, and *Ailurus fulgens*. Among the various potential threats, human activities centered on resource acquisition are the most important, including logging, the collection of medicinal plants, illegal hunting, over grazing, and tourism development. All of these threats are exacerbated by rapid road expansion.

### Protected areas

Our dataset of 159 PAs was collected from the most recent official list of PAs (http://datacenter.mep.gov.cn/), which represents the main parts of the PA system and which was published by China’s Ministry of Environmental Protection [36]. Protected areas in the Hengduan Mountain Hotspot covered some 88,527 km^2^ (or 17.9 percent of the area). According to the protected objects and constructed administrative grades, PAs were divided into national protected areas (NPAs), provincial protected areas (PPAs), and other protected areas (OPAs), which included 17, 49, and 93 PAs, respectively (S1 Table). Other PAs were composed of municipal protected areas and county protected areas. The spatial data of national protected areas were primarily obtained from Satellite Environment Center, Ministry of Environmental Protection (http://www.secmep.cn/secPortal/portal/showpic/showpic1.jsp). The spatial data of provincial protected areas and other protected areas were gathered from the Scientific Survey Report and General Plan of PAs. While these protection status categories differ from the classification standards of the IUCN, they are more applicable to PA management in China. Generally, NPAs appear to be well managed and conserved with better supporting facilities, such as manpower and financial resources, followed by PPAs and OPAs. According to the ecosystem characteristics and key protected targets, PAs were classified into the following 6 types in this region: forest ecosystem protected areas, grassland ecosystem protected areas, wetland ecosystem protected areas, wild animal protected areas, wild plant protected areas, and natural monument protected areas. We merged these into the following four types: forest ecosystem protected areas (FPAs), grassland and wetland ecosystem protected areas (GPAs), wild animal and wild plant protected areas (WPAs), and natural monument protected areas (NMPAs), with 48, 23, 86, and 2 PAs, respectively (S1 Table).

Around each PA, we created buffers at distances of 5 km and 10 km separately outside of the boundaries of the PAs, the range of which were used to reflect the gradient effects on main threats to surrounding areas from the core area of each PA. Considering the issue of overlapping areas between the buffers and PAs, or between different gradient distances of buffers for adjacent PAs, we rectified buffers based on the nearby principle, which classified overlapping areas in relation to corresponding PA or PA buffers with shorter distances. All rectifications were processed using the overlay, extract, and distance analysis tools in the geographic information system (GIS; Arcgis10.0; ESRI).

### Data acquisition and threats analysis

According to the research on threat classification, threats can be divided into two types: direct threats and indirect threats [37]. Indirect threats are usually social, economic, political, or cultural factors, which potentially contribute to and drive direct threats, while direct threats are defined as the proximate human activities that may cause the destruction of ecological resources and processes and degradation or loss of native species and ecological community [14, 37]. Therefore, we used direct threats for quantitative threat assessment in this study. The direct threat indicators are based on a comprehensive list of threats issued by the International Union for Conservation of Nature-Conservation Measures Partnership (IUCN-CMP). A variety of studies have adopted some of these indicators to analyze the negative impacts on PAs [38–41]. Considering the major threat characteristics and data availability of and for the Hengduan Mountain Hotspot, we selected four types of direct threats which were widely used in other research. The threat of residential development is defined as human settlements with a substantial footprint. The threat of agriculture development is defined as threats from agricultural expansion and intensification. The threat of energy production and mining is defined as threats from production of non-biological resources. Other threats, such as transportation and service corridors, biological resource use (e.g., hunting, gathering, and logging), and human intrusions and disturbance (e.g., recreational activities) are related to the accessibility to the threatened areas, and we define these as accessibility threats.

We used high precise land cover and detailed road network data to assess local threats. The land cover database at 1:100000 scale in the Hengduan Mountain Hotspot was updated in 2010 [42, 43]. Remote sensing data include Landsat TM/ETM+ images (http://glovis.usgs.gov/) and HJ-1A/B (http://www.secmep.cn/secPortal/portal/index.faces) were the primary data sources for image interpretation [44, 45]. The land cover classification was based on the geographic object-based image analysis technique [46]. The accuracy of the land cover classification reached 94% [42]. This data with 30 m spatial resolution was more focused on land utilized by human beings than a global land cover database. The threat of residential development was measured by the proxy metric of housing density. We extracted urban and rural settlements from land cover data and calculated the density for every 0.81ha grid cell. The threat of agriculture development was measured by the proxy metric of cultivated land density. We extracted paddy and dry land from land cover data and calculated the density for every 0.81ha grid cell. The threat of energy production and mining was measured by the proxy metric of industrial activity density. We extracted industry land and mining land from land cover data and calculated the density for every 0.81ha grid cell. In order to measure the accessibility threat, we calculated the travel time by considering situations with roads and situations without roads [47]. A vector dataset of road information for this study was obtained from three sources. The road database produced by the National Fundamental Geographic Information Centre in 2005 was the primary data resource, and digitized data from present transportation maps of the provinces of Sichuan, Yunnan, and Tibet, as well as online road maps, were used as supplemental data. There were five different categories of roads: highway, national road, provincial road, county road, and other road (town-village road). We assumed the maximum speeds of different roads according to the road engineering technical standard of China (120, 90, 80, 60, and 30km/h, respectively) (S2 Table) and then converted them to travel times. The travel times in roadless areas were supplemented by simulating the walking speed based on slope data, which was calculated based on a 90m digital elevation model (DEM) from the Shuttle Radar Topography Mission (SRTM) [48, 49]. We used a standard GIS cost-distance algorithm to regard counties as source cells and travel times as costs. Considering that county areas and population might have different contributions to ultimate threats, county areas were divided into four types according to quartiles of the county sizes. We calculated the travel time costs for each type of county, which was weighed by the median population of each type. The population data was collected from China's regional economic statistical yearbook (2010). Finally, we inverted the value of costs so as to cause higher values of travel time costs to have lower threat of accessibility. The following is the equation, where *Cd*_*t*_ and *P*_*t*_ are the values of cost-distance algorithm and median population, respectively, for each type of county area, and *P*_*T*_ is the total population. We calculated this value for each grid cell with the exception of rivers and lakes, which were considered as the obstructed cells and set to no data:

$$\text{Accessibility}=1/\sum_{t=1}^{4}\left[Cd_{t}\text{*}\left(P_{t}/P_{T}\right)\right]$$

To synthesize the four kinds of threats and calculate the local threat value, we first log transformed the value of each kind of threat. Second, by dividing the value of each type of threat by the maximum of each type of threat, respectively, the value of each type of threat was rescaled from 0 to 1. Finally, the values of four kinds of threats were added up for each grid cell and multiplied by 100 [50, 51]. The ultimate result was resampled to the 1ha grid cell.

We calculated the local threat values in different ecoregions, which were defined by World Wildlife Fund (WWF) International [52]. In each ecoregion, we compared the local threat values between protected and unprotected areas. A non-parametric Wilcoxon’s rank sum test was applied to determine if there were significant median differences between local threat values inside PAs and outside PAs [53, 54]. Analysis was conducted in ArcMap 10.0 (ESRI 2010) and R (*wilcox*.*test* function of *stats* package).

We used a conceptual model of integrated threat matrix in order to quantitatively compare the local threats with surrounding threats, which reflect the extent to which local areas are threatened and influenced by surrounding areas [47]. Based on the conceptual model, integrated threat values were divided into six categories. Category 1 was local area with low threats encompassed by low surrounding threats (low-low). Category 2 was local area with low threats encompassed by mixed surrounding threats (low-mixed), which meant surrounding areas had varying threats (low or high) at near and far distances. Category 3 was local area with low threats encompassed by high surrounding threats (low-high). Category 4 was local area with high threats encompassed by low surrounding threats (high-low). Category 5 was local area with high threats encompassed by mixed surrounding threats (high-mixed). Category 6 was local area with high threats encompassed by high surrounding threats (high-high). In order to quantify the surrounding threats, we used a standard GIS rectangular moving-window tool to calculate the threats from surrounding areas. The median value of neighbor window was reassigned to each focal cell. We used the distances of 0.5 km and 10 km from the focal cell to represent the close range and far range threats, respectively. The distances were chosen arbitrarily, but in a manner suitable to reflect the differences.

To divide the grid cells into six threat categories, local threat values were divided into low and high groups based on the median value (Med 1) in the Hengduan Mountain Hotspot. Secondly, for the region with low local threat values, the median values of surrounding threat values for the distances of 0.5 km and 10 km were calculated, respectively (Med 2 and Med 3). Thirdly, for the region with high local threat values, the median values of surrounding threat values of the distances of 0.5 km and 10 km were calculated, respectively (Med 4 and Med 5). We adopted these medians as thresholds to classify the integrated threat values (Table 1).

**Table 1:** Classification criterion of integrated threat categories.

Integrated threat categories	Local threat categories	Classification criterion of local threat categories	Surrounding threat categories	Classification criterion of surrounding threat categories
1	Low	≤Med 1	Low	≤ Med 2, ≤ Med 3
2	Low	≤ Med 1	Mixed	≤ Med 2, ≥ Med 3 or ≥ Med 2, ≤ Med 3
3	Low	≤ Med 1	High	≥ Med 2, ≥ Med 3
4	High	≥Med 1	Low	≤ Med 4, ≤ Med 5
5	High	≥ Med 1	Mixed	≤ Med 4, ≥ Med 5 or ≥ Med 4, ≤ Med 5
6	High	≥ Med 1	High	≥ Med 4, ≥ Med 5

We compared the integrated threat categories among ecoregions and among PAs with different protection levels and with different buffers. To further explore the extent to which main habitats were potentially threatened inside PAs and outside PAs, we overlaid land cover data with the integrated threat categories to analyze the ratios of areas of different threat categories to habitat areas. We considered forest, shrub, grassland, and wetland as four main types of habitats. For each type of habitat, we calculated what proportions of categories 1 and 2 areas PAs contained and unprotected areas contained. Finally, we analyzed the relationships between the sizes of PAs and the proportion of categories 1 and 2 PAs contained, and the proportion of categories 1 and 2 PA buffers contained, respectively.

## Results

### Local threat index

**Fig 2:**
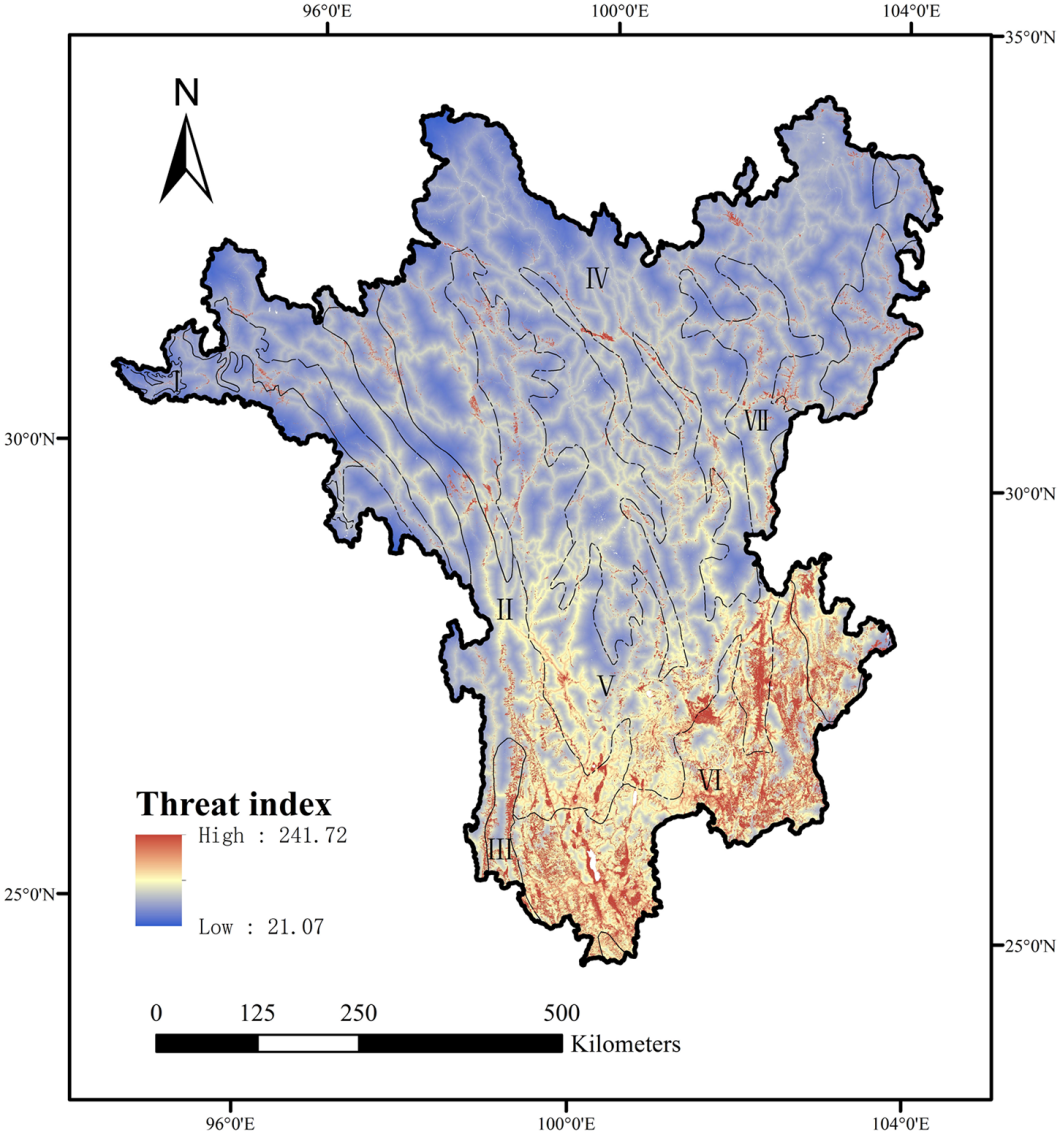
Local threat index calculated by four potential influence factors including residential development, agriculture development, energy production and mining, and accessibility in the Hengduan Mountain Hotspot. The numbers represent seven ecoregions. I: Northeastern Himalayan subalpine conifer forests (NHSCF), II: Nujiang Langcang Gorge alpine conifer and mixed forests (NLCMF), III: Northern Indochina subtropical forests (NISF), IV: Southeast Tibet shrublands and meadows (STSM); V: Hengduan Mountains subalpine conifer forests (HMSCF); VI: Yunnan Plateau subtropical evergreen forests (YPSEF); VII: Qionglai-Minshan conifer forests (QMCF).

The spatial variation in the values of the local threat index was quite notable from south to north for the Hengduan Mountain Hotspot. Higher threat values were obviously centralized in distribution in the southern region, which represented the diverse and intense threats, while threats in the central-northern area were relatively weaker with band-shaped distribution, meaning the relative homogeneous threats were mainly caused by roads and agriculture development along the roads (Fig 2). There were seven main terrestrial ecoregions in this region (S3 Table). The most seriously threatened region was concentrated in the region of Yunnan Plateau subtropical evergreen forests (YPSEF). The median value of threat index was 70.12, which was mainly caused by high-density agriculture development, growing residential development, and transport corridor threats. Local threat value in the region of Northern Indochina subtropical forests (NISF) ranked second, the median value of which was 60.88. The major effect factors were agriculture development and transport corridor threats. The region of Hengduan Mountains subalpine conifer forests (HMSCF) was the core area of the Hengduan Mountains. The median value of threat index was 52.88. The region of Southeast Tibet shrublands and meadows (STSM), which accounted for 43.4% of the total area in the Hengduan Mountain Hotspot, was at a relatively lower risk.

**Fig 3:**
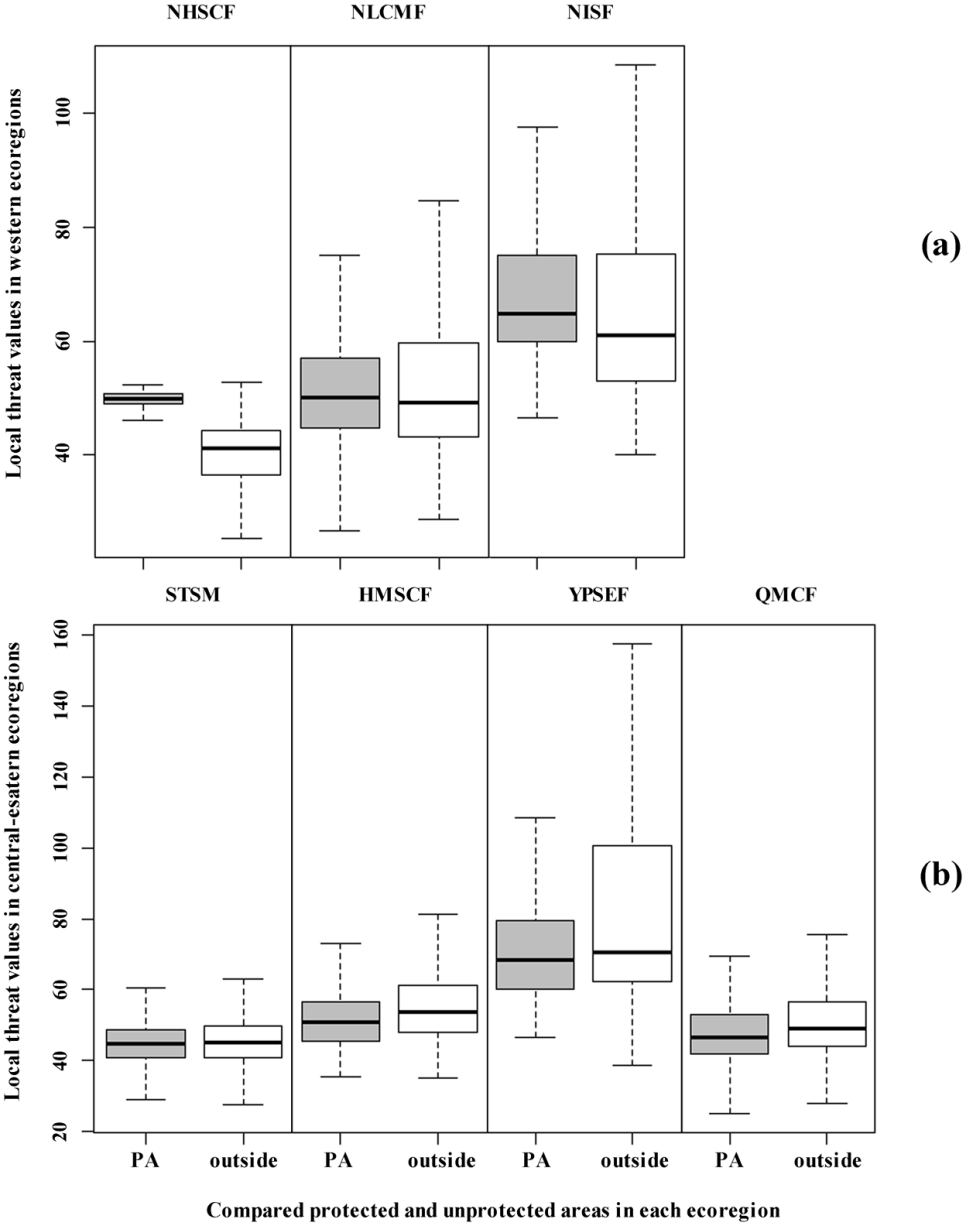
Boxplot summaries of the values of local threat index inside protected areas and outside protected areas of seven ecoregions, including three western ecoregions (a) and four central-eastern ecoregions (b). Boxes shaded in gray indicate that the value of local threat index was significantly different between inside protected areas and outside protected areas for each ecoregion, based on a Wilcoxon rank sum test at *P* value ≤ 0.05. The bold lateral bars represent the median values of local threat index, the boxes represent the first and third quantiles, and the whiskers depict the range of the data.

Nonparametric test supported the conclusion that the median difference in values of local threat index was significantly different between paired areas inside PAs and areas outside PAs in each ecoregion (p<0.01) (Fig 3). For the three western ecoregions, median values of local threat index inside PAs were higher than values outside PAs. Because the PA areas in the region of Northeastern Himalayan subalpine conifer forests (NHSCF) were small (S3 Table), the distribution of the values of local threat index was relatively concentrated. For the four central-eastern ecoregions, median values of local threat index inside PAs were lower than values outside PAs. In the region of YPSEF, the dispersion degree of the values of local threat index both inside and outside PAs was higher than the dispersion degree in the regions of STSM, HMSCF, and Qionglai-Minshan conifer forests (QMCF), which indicated that the region of YPSEF contained more diverse and serious threats.

### Integrated threat categories

**Fig 4:**
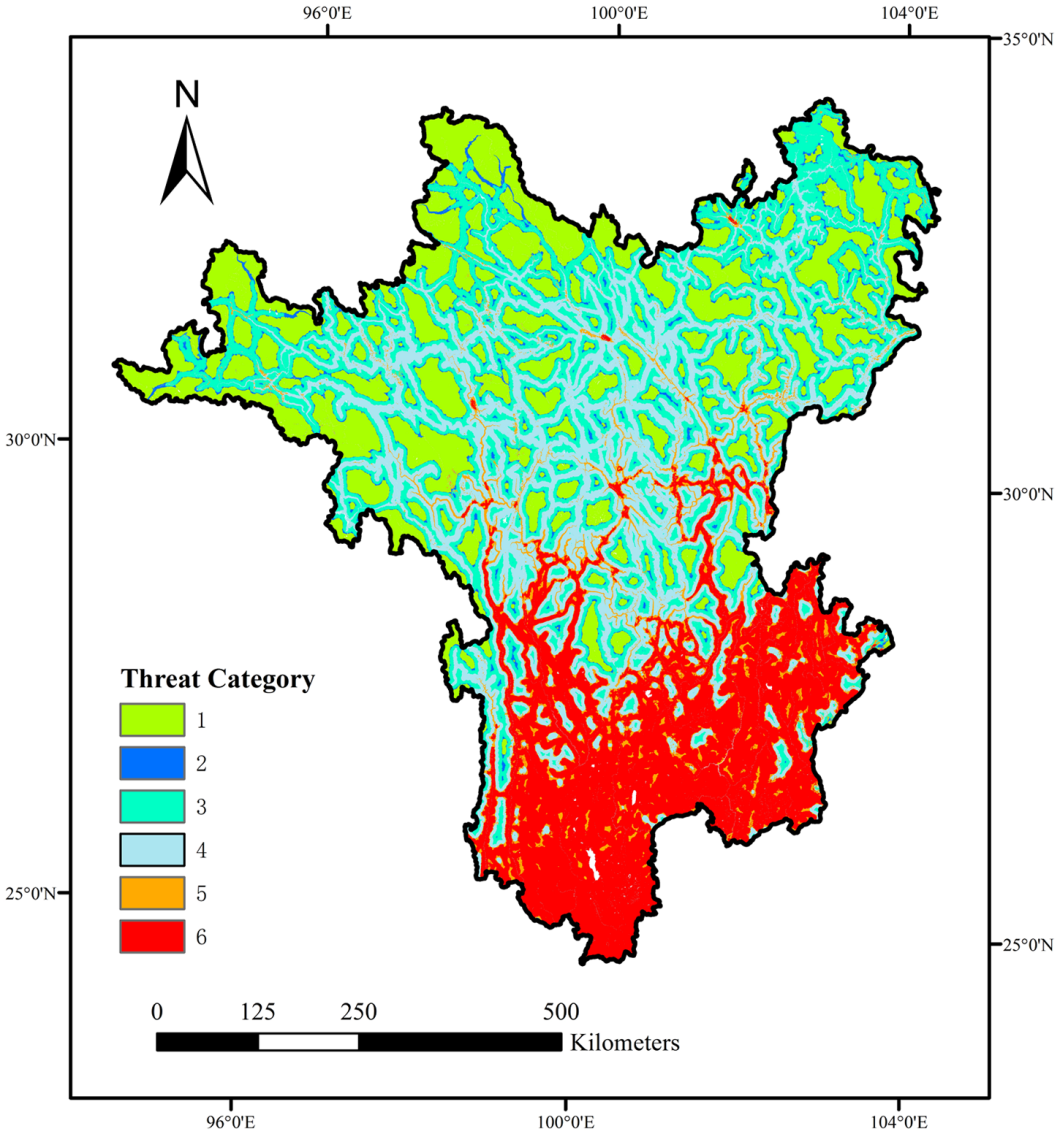
Integrated threat categories mapped in the Hengduan Mountain Hotspot considering both the local threat index and surrounding threat index.

Categories 1 and 2, which indicated low local threat encompassed by relatively unthreatened surrounding areas, could be considered the core areas of conservation areas. They were mainly distributed in northern areas with large sizes and tended to be small patches in southern areas (Fig 4). The region of STSM comprising 43.9% of the land area of the Hengduan Mountain Hotspot was the largest ecoregion (S4 Table). It contained 40.4% of its land area in categories 1 and 2, and was the least threatened ecoregion. Although the region of NHSCF contained 71.7% of its area in categories 1 and 2, it comprised less than 1% of the land area of the Hengduan Mountain Hotspot. Categories 4 through 6, which indicated high local threat levels, were the areas of high risk from intensive human activities. For instance, the southern regions of YPSEF and NISF contained 86.9% and 61.9% of their area in category 6, respectively. The central regions of HMSCF, NLCMF, and QMCF contained only 30.7%, 25.5%, and 18% of their area in category 6, respectively. However, the proportion reached 70.3%, 52.3%, and 47.7%, respectively, when summing up these categories from categories 4 through 6.

For all areas of categories 1 and 2 in the Hengduan Mountain Hotspot, 22.4% of which was contained in PAs (S5 Table). Considering the proportions of different types of habitats, forest habitats (26.8%) and wetland habitats (66.3%) had more categories 1 and 2 areas contained in PAs, which meant PAs conserved more core areas of wetland and forest habitats. Generally, lands with higher PA coverage were more likely to have higher proportions of core areas covered by PAs (S3 and S5 Tables). For instance, the region of QMCF with 24.8% PA coverage contained the highest ratio of core areas in PAs (33.7%). The ratio of core areas in this region was also highest for forests (34.6%), shrubs (35.2%), and grasslands (29.0%). The region of STSM with 24.9% PA coverage contained the second highest ratio of core areas in PAs (25.7%), but it had the highest ratio of core areas covered by PAs for wetlands (67.1%). Compared with the ratios of core area to land area outside PAs, the ratios of core area to land area inside PAs were higher for all four types of habitats in the regions of QMCF and STSM, which meant PAs were more effective in these regions. In the region of HMSCF, with only 7.4% PA coverage, the ratio of core area to land area inside PAs was 6.6% higher than the ratio outside PAs. However, in the region of NLCMF, with 15.4% PA coverage, the ratio of core area to land area inside PAs was 7.6% lower than the ratio outside PAs, because the ratios of core area to land area inside PAs were lower for shrubs and grasslands. Furthermore, there was no core area of wetlands inside PAs.

### Protected areas with gradient buffer zones

**Fig 5:**
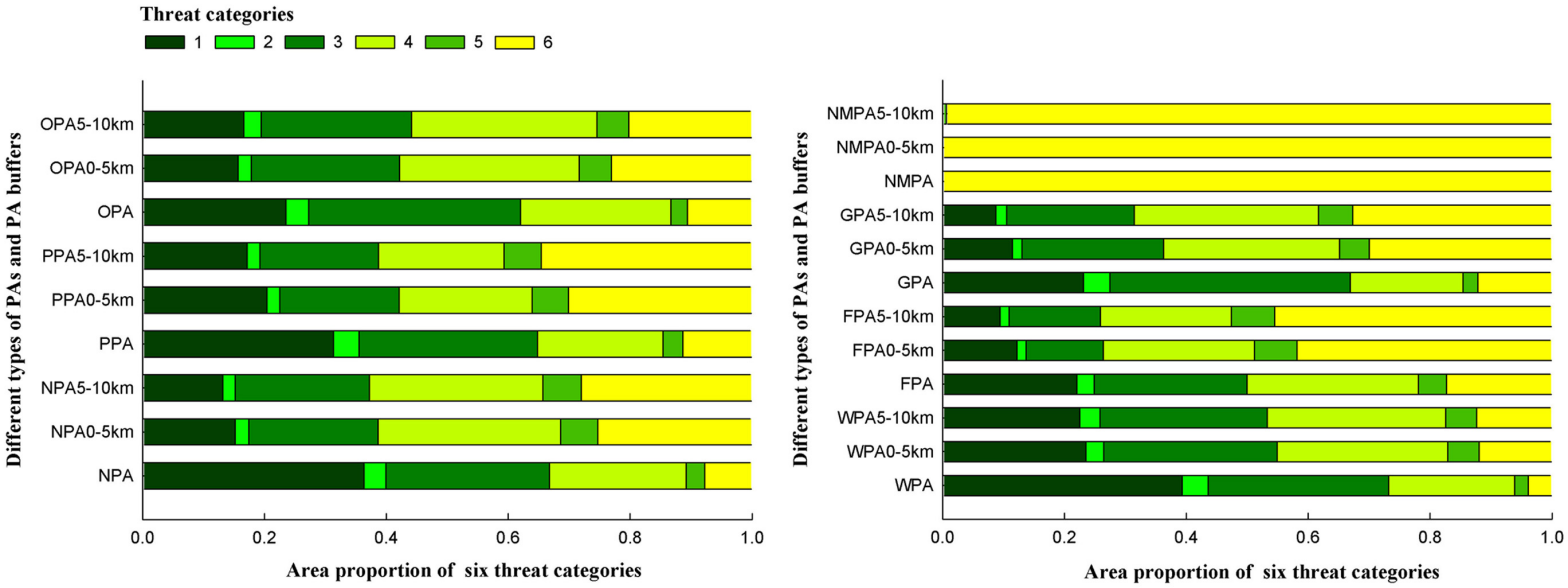
Proportions of six integrated threat categories contained in PAs and PA buffers based on three kinds of protection levels for PAs shown on the left, and four kinds of ecosystem characteristics and protected targets for PAs shown on the right; 0–5 km and 5–10 km represent PA buffers at 5-km intervals.

By calculating the average value of local threat index in every single protected area, we found the lowest threat levels were in national protected areas (NPAs), followed by provincial protected areas (PPAs). Both NPAs and PPAs had significantly lower threat levels than the other protected areas (OPAs) (p<0.05). Integrated threat categories of different types of PAs demonstrated similar results (Fig 5). NPAs contained the highest proportion in category 1(36.4%) compared with PPAs and OPAs (31.3% and 23.5%, respectively). However, when considering categories 1 through 3, the proportions were nearly equal for the three types of PAs, which indicated PPAs and OPAs had smaller core areas with relatively high risk from surrounding areas.

The results differed considerably depending on the categories of protected areas based on the classification of different ecosystem characteristics and key protected targets. Wild animal and wild plant protected areas (WPAs) had the lowest local threat index, which was significantly lower than the local threat index for both forest ecosystem protected areas (FPAs) and grassland and wetland ecosystem protected areas (GPAs) (p<0.01). The average value for the local threat index of GPAs was lower than the average value for FPAs, but not significantly. The average value for natural monument protected areas (NMPAs) was highest, however, without any consideration given to statistical tests due to a low number of samples. Integrated threat categories indicated that WPAs contained a higher proportion in category 1(39.3%) than the other three types of PAs. When considering categories 1 through 3, the proportion reached 73.2%. Although GPAs and FPAs contained a similar proportion in category 1, GPAs contained 66.9% of their area in categories 1 through 3, approximately 17% higher than the percentage of area for FPAs. NMPAs contained no core areas of habitats inside PAs, and most of their surrounding areas were regions of intensive human activities.

The proportion of integrated threat categories in buffer zones of 5-km and 10-km revealed ways in which the threats changed from PAs within the borders to surrounding areas far from the borders. For most PAs, the proportion of integrated threat categories 1 through 3 declined from PAs within the borders to buffer zones 5-km outside PAs, and to buffer zones 10-km outside PAs (Fig 5). It is worth noting that the dramatic declines were mainly concentrated in buffer zones 0–5 km outside PAs, and varying slightly in buffer zones 5–10 km outside PAs. However, national protected areas suffered more external threats in 5-km buffers outside PAs than other types of PAs. The proportion of category 1 areas in 5-km buffers outside the borders of NPAs dropped by 58.3% compared to the proportion within NPAs, while PPAs and OPAs dropped by 34.8% and 33.4%, respectively. The proportion of categories 1 through 3 in 5-km buffers outside NPAs reached 38.7%, while PPAs and OPAs reached 42.1% and 42.2%, respectively. WPAs suffered less external threats outside PAs than FPAs and GPAs. Although the proportion of category 1 areas in 5-km buffers outside WPAs evidently decreased compared to the proportion of category 1 areas within WPAs, 23.5% of the area in the 5-km buffers were still covered by category 1. When considering category areas 1 through 3, the proportion in 5-km buffers outside WPAs only dropped by 25.0%, while the proportion of FPAs and GPAs dropped by 47.2% and 45.8%, respectively.

**Fig 6:**
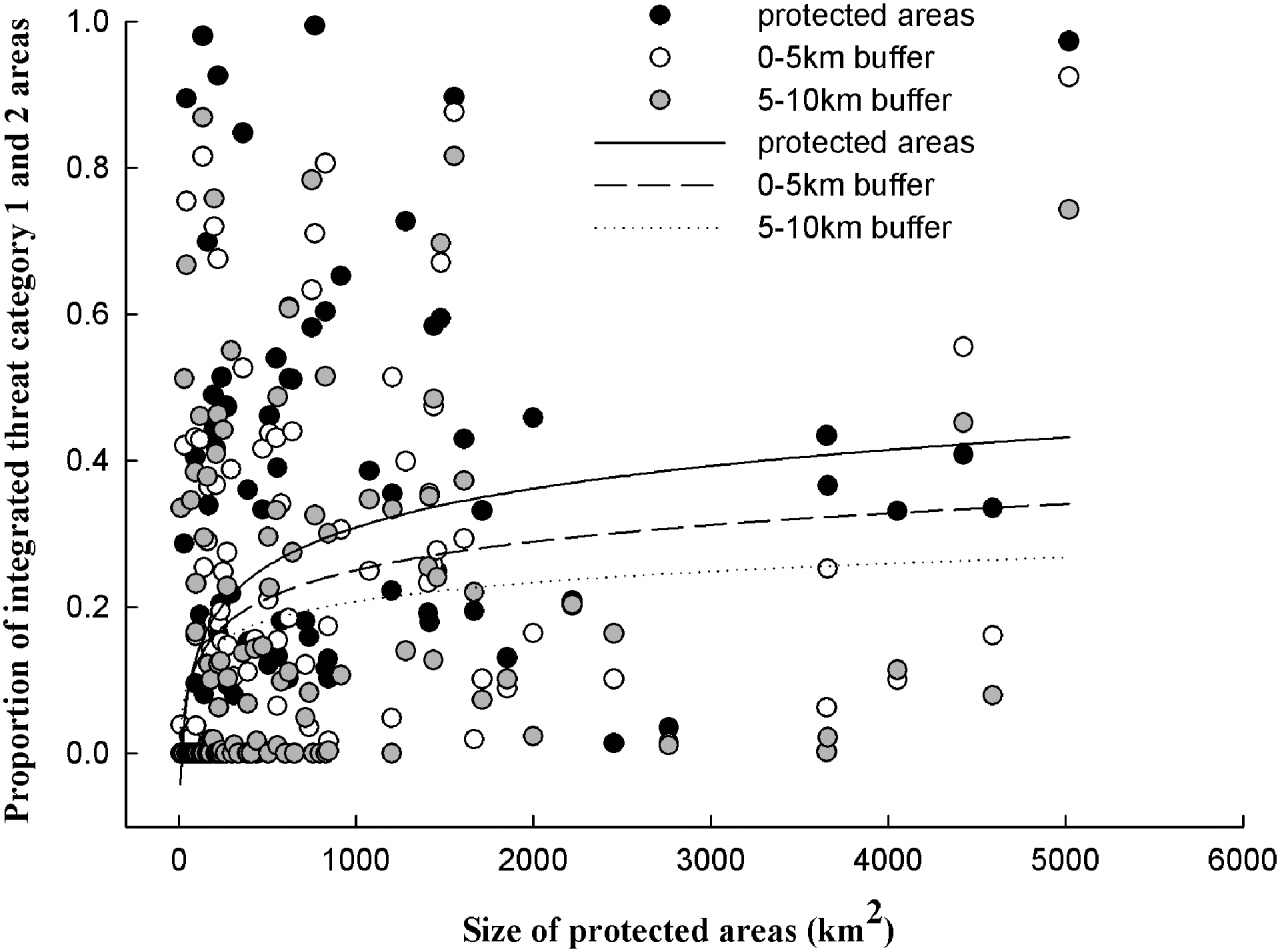
The relationship between the size of protected areas and the proportion of integrated threat category 1 and 2 areas that PAs and PA buffers contain.

To reveal whether PAs with larger areas might face lower risk from external environmental threats, every single PA within the borders, in buffer zones 0–5 km outside PAs, and in buffer zones 5–10 km outside PAs were calculated separately to evaluate the proportion of integrated threat category 1 and 2 areas that they contained. We removed PAs less than 1km^2^ in size to ensure that every PA contained enough threat category pixels. The results of a logarithmic fit suggested a positively significant correlation between the size of protected areas and the proportion of integrated threat category 1 and 2 areas that they contained inside PAs (R^2^ = 0.13, p<0.01) (Fig 6). However, the positive correlations gradually weakened as buffer zones’ distance increased. In addition, a positive correlation was found between the size of protected areas and the proportion of integrated threat category 1 and 2 areas that they contained in buffer zones 0–5 km outside PAs (R^2^ = 0.09, p<0.01), and a much weaker positive correlation was found between the size of protected areas and the proportion of integrated threat category 1 and 2 areas that they contained in buffer zones 5–10 km outside PAs (R^2^ = 0.05, p<0.05).

## Discussion

### Protected areas and unprotected areas

According to a worldwide meta-analysis, PAs generally have higher abundances of individual species and higher species richness values compared with surrounding areas [55]. Therefore, if these PAs are less threatened, we can consider biodiversity-abundant regions to be safe. This study shows that the local threat index differs significantly between paired areas inside PAs and areas outside PAs in each ecoregion. Most areas have a lower threat inside PAs than outside of PAs. A study of 180 selected PAs in China had similar results, finding the change rates of land cover from 1980s to 2005 inside PAs to be lower than the rates outside of PAs [56]. The median values of local threat index are higher inside PAs for three western ecoregions. For the regions of NHSCF and NISF, this is probably because the PA areas are small, which makes it easy to be affected by surrounding threats (S3 Table). For the region of NLCMF, the reason may be the uneven distribution of PAs, most of which are distributed in the southern region with low altitudes and relatively high population densities. While in the northern region with low threat levels, there were a low number of PAs. Overall, we find that PAs in the Hengduan Mountain Hotspot are further away from the main threat than unprotected lands. The location of PAs may be an important cause of the differences in threats between PAs and unprotected areas. One study found that deforestation rates within PAs were approximately 2.7% lower than the deforestation rates on all unprotected lands in Acre, Brazil [57]. However, when removing the influences of location characteristic differences, there were no significant differences. Another study found that deforestation was much higher near roads and rivers than elsewhere in the Amazon, but even when these forests were accessible, PAs showed resistance to deforestation compared to unprotected forests [40]. Hence, the differences between ecological influences inside PAs and outside PAs are determined by both the locations of PAs and PA management. This threat analysis can provide a reference for crucial habitat conservation and establishment of new PAs in the future.

### PA management for local and surrounding threats

This study demonstrates that NPAs have lower threat values than PPAs and OPAs, suggesting that PAs with higher levels of protection management are relatively less threatened. Although these categories are different from the classification standards used worldwide, NPAs with comprehensive protection planning and fixed funding resources are considered to be strict PAs, similar to the GAP stewardship levels 1 and 2 in the US or integral protection in Brazil. The potential ecological impacts caused by differences in management levels have been proved. Pfaff et al. [57] found that deforestation in protected areas is lowest for the category of integral protection and highest for the category of sustainable use protection. Barber et al. [40] found federal strict PAs had the lowest levels of deforestation, while state-managed PAs with high clearing threat had the highest levels of deforestation.

Although the Hengduan Mountain Hotspot is rich in biodiversity with relatively low population, the obviously increasing proportion of integrated threat categories 4–6 outside PAs indicates that surrounding areas are more seriously threatened than the PAs. The main reason for this is that PAs do not encourage land conservation nearby as we expected [58]. On the contrary, since PAs can provide increasing ecosystem services and scarce resources for inhabitants in rural areas, they may attract immigrants and human settlement to PA borders [23]. Population growth and housing density increases around PAs have significant negative impacts on biodiversity [10, 23, 59]. Although litter data are available to analyze the population levels around PAs in this study, higher threat values around PAs in the Hengduan Mountain Hotspot follow similar patterns in that human activities are mostly concentrated in the areas surrounding PAs. A study of 180 NPAs in China also indicated that the landscape pattern of land cover in the surrounding areas was less stable than that in PAs [56].

We find that dramatic declines of core areas are mainly concentrated in the buffer zones of 0–5 km outside PAs. This finding means that PAs do not even play a role in gradually mitigating the threats in PA buffers. On the contrary, human activities directly concentrated around the borders, which reflect evident edge effect. The main reason for this is that although PA borders are effective in resisting human interference, PAs are resource-abundant regions in the Hengduan Mountain Hotspot, in which people tend to log, collect plants, and hunt for economic interests and traditional medicinal purposes. The distance of 5-km may be an important threshold bringing serious ecological impacts [40]. For instance, declines in the proportion of forest areas were mainly concentrated in 5-km buffers outside three Indian PAs [60]. However, NPAs contain a low proportion of core areas in 5-km buffers compared to PPAs and OPAs in the Hengduan Mountain Hotspot. High levels of PA management have little effect in surrounding areas.

### Efficacy of PA sizes

Large PAs contain more unthreatened lands in the surrounding areas than small PAs do. We use the proportion of core areas in PA buffers to analyze the relationship rather than the size of core areas in PA buffers, which has the effect of making the results more reasonable. The size of each buffer is not necessarily proportional to the size of the corresponding PA due to the buffer rectification we mentioned in materials and method. Generally, for large PAs, challenges and conservation objectives lie primarily in managing land use change in order to minimize impacts to PAs within the borders [61]. For small PAs, conservation objectives are focused on preserving remaining and crucial habitats and providing connectivity to regional PA networks. We are unable to explain why large PAs can contain a high proportion of core areas in PAs or PA buffers. Perhaps large PAs are less likely to be impacted by human activities, or perhaps large PAs tend to be surrounded by relatively safe environments when the PAs are established. Therefore, more long time series studies are needed to analyze the changes and explain the differences. Regardless, the results can serve PA managers in taking measures for effective conservation based on the different PA sizes. For large PAs, main habitats potentially threatened and underrepresented in categories 1 and 2 should receive greater attention with regard to enlarging core habitat areas. For small PAs, core areas and integrated threat category 3 should be identified as the most important ecological corridors to enhance protection and restoration so as to maintain connectivity.

### Uncertainties involved in threat assessment

We present a comprehensively quantitative evaluation of potential threats to the current PA network and their buffers at a regional scale in the Hengduan Mountain Hotspot, which is seldom studied. However, threat assessment is always accompanied by the uncertainty arising from errors in data [62], incomplete consideration of the various sources of threats, or uncontrollable stochastic factors [14]. Certain differences are found between PA boundary data produced by the Ministry of Environmental Protection and that produced by local conservation planning, especially with regard to municipal PAs and county PAs. In addition, not all PA management strategies are strictly implemented based on the boundaries. Theoretical conservation areas cannot coincide exactly with the actual management range due to ambiguous boundaries. The results of the threat analysis may produce certain errors for PAs near the border of the Hengduan Mountain Hotspot. For instance, we use the cost-distance algorithm for the accessibility threat analysis, which takes urban areas within the border as the primary resource input. However, this method would underestimate the threat value, as urban areas and roads beyond the border might also increase the accessibility threat, especially in the east and south of the region. The sources of threats that we considered were incomplete in this study. Many potential threats, including hunting, gathering and logging, were indirectly covered using accessibility analysis. Nevertheless, errors may exist in this analysis due to the uncertainty and randomicity of threats that are caused by selective activities. Dams are undoubtedly another major challenge in ecological protection. Since it is difficult to obtain detailed data on dams, we were unable to adequately quantify such data in this analysis. Other unpredictable factors, including stochastic disturbances (e.g., grazing and land conversion), geological events, and severe weather events may also affect the current threat assessment; these are not reflected in our threat analysis. In addition, four kinds of threat weights are the same in the analysis. Nevertheless, we think different weights are more valuable for local scale studies.

### Suggestions for PA management

Approximately 22.4% of the area of integrated threat categories 1 and 2 is covered by PAs, which is higher than the proportion of PA coverage (17.9%) in the Hengduan Mountain Hotspot. Although more than 20% of the area of NPAs, PPAs, and OPAs is contained in categories 1 and 2, respectively, in the Hengduan Mountain Hotspot, it is worth noting that many PAs are distributed in higher elevation mountain areas. Integrated threat categories 1 and 2 in PAs may not refer to the vegetation but rather to the snow cover area or bare area. Therefore, the actual core areas of habitats were smaller (e.g., HMSCF 65%) (S5 Table). Nevertheless, this assessment shows how severe threat values are spatially distributed outside of PAs compared with those inside PAs. The administrative departments that oversee PAs may use these results coupled with land cover data to monitor, analyze, and implement management strategies for maintaining the effectiveness of regional PA networks. This method will be even more valuable for sustainable PA management at regional or local scales with up-to-date, highly precision data.

On the basis of their understanding of threats, such as integrated threat categories, managers can take appropriate measures to enhance objective management and improve efficiency based on different threat categories [63]. For example, habitats of categories 1 and 2, which are considered to be core areas, should be most important in landscape conservation. Habitats of category 3 with core areas surrounded by high threats can be regarded as islands of biodiversity conservation [47]. In order to link these isolated habitats with other habitats of category 1 and category 3 between two separate PAs or among more PAs within a PA network, more corridors (e.g., habitats of category 2) should be protected and established to maintain connectivity for wildlife, especially in important wild animal and wild plant PAs (WPAs), such as the Wolong Natural Reserve and the Jiuzhaigou Valley Natural Reserve. Category 4 encompasses seriously threatened areas close to core areas of habitats. Threats to the closest neighboring areas should be given special attention and be strictly monitored, as these threats are often the most likely causes of ecological degradation in remaining habitats; their appropriate management will result in clear improvements in ecological conditions. It is also critical to link the threat assessment with something biologically meaningful to managers [64, 65]. Because endemic species differ in their home ranges, movement, and dispersal capabilities, the fact that the distance between surrounding threats and the core area of habitat is small will have adverse impacts on certain species.

Overall, transferring development activities to areas more distant from PAs is an essential approach to ecological protection [66]. However, future land-use change around PAs is inevitable, and require serious consideration of potential threats to safeguard the conservation value of PAs and to mitigate degradation [8, 67]. Threat assessments must be conducted prior to implementing any new construction project within and outside of PAs.

## Supporting Information

S1 Table Statistics of different types of PAs in the Hengduan Mountain Hotspot. (DOCX)

S2 Table Maximum speeds of different types of road according to road engineering technique standards of China. (DOCX)

S3 Table Statistics of seven main ecoregions covered by PAs in the Hengduan Mountain Hotspot. (DOCX)

S4 Table Ratio of integrated threat category area each ecoregion contain. (DOCX)

S5 Table Proportion of category 1 and 2 areas of habitat to habitat areas for each region. (DOCX)

## Abbreviations

FPAs: forest ecosystem protected areas
GPAs: grassland and wetland ecosystem protected areas
HMSCF: Hengduan Mountains subalpine conifer forests
NHSCF: Northeastern Himalayan subalpine conifer forests
NISF: Northern Indochina subtropical forests
NLCMF: Nujiang Langcang Gorge alpine conifer and mixed forests
NMPAs: natural monument protected areas
NPAs: national protected areas
OPAs: other protected areas
PAs: protected areas
PPAs: provincial protected areas
QMCF: Qionglai-Minshan conifer forests
STSM: Southeast Tibet shrublands and meadows
WPAs: wild animal and wild plant protected areas
YPSEF: Yunnan Plateau subtropical evergreen forests
