# Supplementary material for: Assessing Local and Surrounding Threats to the Protected Area Network in a Biodiversity Hotspot: The Hengduan Mountains of Southwest China
Source: PLoS One. 2015 Sep 18;10(9):e0138533. doi: 10.1371/journal.pone.0138533 (PMC4575193; doi:10.1371/journal.pone.0138533)
Supplement: S1 Table — (DOCX) [file pone.0138533.s001.docx]

**S1 Table. Statistics of different types of PAs in the Hengduan Mountain Hotspot.**

| **Types of PAs** | **FPAs** | | **GPAs** | | **WPAs** | | **NMPAs** | | **Total** | |
| --- | --- | --- | --- | --- | --- | --- | --- | --- | --- | --- |
|  | **Number** | **Area (km^2^)** | **Number** | **Area (km^2^)** | **Number** | **Area (km^2^)** | **Number** | **Area (km^2^)** | **Number** | **Area (km^2^)** |
| NPAs | 5 | 12582.88 | 3 | 7054.32 | 9 | 14917.50 | 0 | 0 | 17 | 34554.70 |
| PPAs | 16 | 9429.65 | 7 | 5175.71 | 26 | 11072.61 | 0 | 0 | 49 | 25677.97 |
| OPAs | 27 | 5118.25 | 13 | 8094.63 | 51 | 15068.89 | 2 | 13.00 | 93 | 28294.77 |
| Total | 48 | 27130.78 | 23 | 20324.66 | 86 | 41059.00 | 2 | 13.00 | 159 | 88527.44 |

NPAs, national protected areas; PPAs, provincial protected areas; OPAs, other protected areas composed by municipal protected areas and county protected areas; FPAs, forest ecosystem protected areas; GPAs, grassland and wetland ecosystem protected areas; WPAs, wild animal and wild plant protected areas; NMPAs, natural monument protected areas
