# Supplementary material for: Assessing Local and Surrounding Threats to the Protected Area Network in a Biodiversity Hotspot: The Hengduan Mountains of Southwest China
Source: PLoS One. 2015 Sep 18;10(9):e0138533. doi: 10.1371/journal.pone.0138533 (PMC4575193; doi:10.1371/journal.pone.0138533)
Supplement: S2 Table — (DOCX) [file pone.0138533.s002.docx]

**S2 Table.** **Maximum speeds of different types of road according to road engineering technique standards of China.**

| **Road type** | **Speed(km/h)** | **Travel time(min/km)** |
| --- | --- | --- |
| highway | 120 | 0.5 |
| National road | 90 | 0.67 |
| Provincial road | 80 | 0.75 |
| County road | 60 | 1 |
| Other road | 30 | 2 |
| No roads | 6*exp[-3.5*abs(slope+0.05)] | 10/exp[-3.5*abs(slope+0.05)] |
