# Supplementary material for: Assessing Local and Surrounding Threats to the Protected Area Network in a Biodiversity Hotspot: The Hengduan Mountains of Southwest China
Source: PLoS One. 2015 Sep 18;10(9):e0138533. doi: 10.1371/journal.pone.0138533 (PMC4575193; doi:10.1371/journal.pone.0138533)
Supplement: S3 Table — (DOCX) [file pone.0138533.s003.docx]

**S3 Table.** **Statistics of seven main ecoregions covered by PAs in the Hengduan Mountain Hotspot.**

| **Ecoregion** | **Area (km^2^)** | **PA area (km^2^)** | **Ratio of PA area (%)** |
| --- | --- | --- | --- |
| NHSCF | 2080.42 | 44.56 | 2.14 |
| NLCMF | 60277.4 | 9302.95 | 15.43 |
| NISF | 6830.82 | 238.03 | 3.48 |
| STSM | 214115 | 53306.24 | 24.9 |
| HMSCF | 99147 | 7329.64 | 7.39 |
| YPSEF | 57600.3 | 2848.81 | 4.95 |
| QMCF | 47516.2 | 11766.7 | 24.76 |

NHSCF, Northeastern Himalayan subalpine conifer forests; NLCMF, Nujiang Langcang Gorge alpine conifer and mixed forests; NISF, Northern Indochina subtropical forests; STSM, Southeast Tibet shrublands and meadows; HMSCF, Hengduan Mountains subalpine conifer forests; YPSEF, Yunnan Plateau subtropical evergreen forests; QMCF, Qionglai-Minshan conifer forests
