# Supplementary material for: Assessing Local and Surrounding Threats to the Protected Area Network in a Biodiversity Hotspot: The Hengduan Mountains of Southwest China
Source: PLoS One. 2015 Sep 18;10(9):e0138533. doi: 10.1371/journal.pone.0138533 (PMC4575193; doi:10.1371/journal.pone.0138533)
Supplement: S4 Table — (DOCX) [file pone.0138533.s004.docx]

**S4 Table.** **Ratio of integrated threat category area each ecoregion contain.**

| **Ecoregion** | **Category1 (%)** | **Category2 (%)** | **Category3 (%)** | **Category4 (%)** | **Category5 (%)** | **Category6 (%)** | **Category1&2 (%)** | **Category4 to 6 (%)** |
| --- | --- | --- | --- | --- | --- | --- | --- | --- |
| NHSCF | 63.29 | 8.45 | 20.42 | 7.66 | 0.18 | 0 | 71.74 | 7.84 |
| NLCMF | 22.63 | 2.82 | 22.27 | 22.05 | 4.72 | 25.51 | 25.45 | 52.28 |
| NISF | 1.38 | 1.13 | 12.15 | 15.04 | 8.44 | 61.86 | 2.51 | 85.34 |
| STSM | 36.27 | 4.08 | 31.71 | 23.75 | 2.48 | 1.71 | 40.35 | 27.94 |
| HMSCF | 9.44 | 1.69 | 18.53 | 32.1 | 7.5 | 30.74 | 11.13 | 70.34 |
| YPSEF | 0.05 | 0.04 | 1.53 | 5.32 | 6.18 | 86.88 | 0.09 | 98.38 |
| QMCF | 21.62 | 3.46 | 27.23 | 23.95 | 5.74 | 18 | 25.08 | 47.69 |

NHSCF, Northeastern Himalayan subalpine conifer forests; NLCMF, Nujiang Langcang Gorge alpine conifer and mixed forests; NISF, Northern Indochina subtropical forests; STSM, Southeast Tibet shrublands and meadows; HMSCF, Hengduan Mountains subalpine conifer forests; YPSEF, Yunnan Plateau subtropical evergreen forests; QMCF, Qionglai-Minshan conifer forests
