# Supplementary material for: Assessing Local and Surrounding Threats to the Protected Area Network in a Biodiversity Hotspot: The Hengduan Mountains of Southwest China
Source: PLoS One. 2015 Sep 18;10(9):e0138533. doi: 10.1371/journal.pone.0138533 (PMC4575193; doi:10.1371/journal.pone.0138533)
Supplement: S5 Table — (DOCX) [file pone.0138533.s005.docx]

**S5 Table.** **Proportion of category 1 and 2 areas of habitat to habitat areas for each region.**

| **Region** | **Total habitat** | | | | **Forest habitat** | | | **Shrub habitat** | | | **Grassland habitat** | | | **Wetland habitat** | | |
| --- | --- | --- | --- | --- | --- | --- | --- | --- | --- | --- | --- | --- | --- | --- | --- | --- |
|  | **PCI (%)** | **PCO (%)** | **CCP (%)** | **PCH (%)** | **PCI (%)** | **PCO (%)** | **CCP (%)** | **PCI (%)** | **PCO (%)** | **CCP (%)** | **PCI (%)** | **PCO (%)** | **CCP (%)** | **PCI (%)** | **PCO (%)** | **CCP (%)** |
| NHSCF | 0.66 | 73.07 | 0.02 | 92.00 | 0 | 87.63 | 0 | 0 | 73.68 | 0 | 1.89 | 70.44 | 0.06 | 0 | 67.12 | 0 |
| NLCMF | 19.05 | 26.61 | 11.57 | 85.02 | 14.53 | 11.02 | 26.78 | 19.01 | 27.01 | 9.44 | 26.67 | 35.88 | 8.3 | 0 | 13.87 | 0 |
| NISF | 0 | 2.6 | 0 | — | 0 | 2.28 | 0 | 0 | 1.54 | 0 | 0 | 4.43 | 0 | — | 80.95 | 0 |
| STSM | 41.59 | 39.94 | 25.65 | 78.28 | 27.52 | 20.78 | 32.66 | 36.2 | 30.46 | 25.43 | 41.21 | 40.52 | 24.18 | 48.62 | 35.64 | 67.13 |
| HMSCF | 17.27 | 10.65 | 11.39 | 65.00 | 5.68 | 6.56 | 5.11 | 12.37 | 9.81 | 8.59 | 22.68 | 16.28 | 11.53 | 10.63 | 11.67 | 15.93 |
| YPSEF | 0 | 0.1 | 0 | — | 0 | 0.04 | 0 | 0 | 0.22 | 0 | 0 | 0.03 | 0 | 0 | 0 | — |
| QMCF | 34.03 | 22.13 | 33.68 | 79.15 | 24.04 | 15.11 | 34.6 | 28.7 | 15.69 | 35.16 | 51.58 | 43.96 | 29.01 | 45.62 | 42.47 | 16.89 |
| HMH |  |  | 22.43 |  |  |  | 26.82 |  |  | 22.13 |  |  | 21.39 |  |  | 66.32 |

NHSCF, Northeastern Himalayan subalpine conifer forests; NLCMF, Nujiang Langcang Gorge alpine conifer and mixed forests; NISF, Northern Indochina subtropical forests; STSM, Southeast Tibet shrublands and meadows; HMSCF, Hengduan Mountains subalpine conifer forests; YPSEF, Yunnan Plateau subtropical evergreen forests; QMCF, Qionglai-Minshan conifer forests; HMH, the Hengduan Mountain Hotspot; PCI, proportion of category 1 and 2 areas of habitat to habitat areas in PAs; PCO, proportion of category 1 and 2 areas of habitat to habitat areas outside PAs; CCP, category 1 and 2 areas of habitat covered by PAs; PCH, proportion of category 1 and 2 areas of habitat to category 1 and 2 areas in PAs.
